# Incidence of Gallbladder Carcinoma: Conservative or Radical Surgery

**DOI:** 10.1155/1993/42150

**Published:** 1993

**Authors:** A. N. Kingsnorth

**Affiliations:** Department of Surgery The University of Liverpool, P O Box 147 Liverpool L69 3BX UK


*HPB Surgery*, 1993, Vol. 7, pp. 175–184
Reprints available directly from the publisher
Photocopying permitted by license only

© 1993 Harwood Academic Publishers GmbH
Printed in the United States of America

### HPB INTERNATIONAL

**EDITORIAL & ABSTRACTING SERVICE**

JOHN TERBLANCHE, EDITOR
Department of Surgery,
Medical School · Observatory 7925
Cape Town · South Africa
Telephone: (021) 47-1250 Ext. 2
Telefax (021) 448-6461

### INCIDENCE OF GALLBLADDER CARCINOMA: CONSERVATIVE OR RADICAL SURGERY

### ABSTRACTS

*Shirai, Y., Yoshida, K., Tsukada, K. and Muto, T. (1992) Inapparent carcinoma of the gallbladder. Annals of Surgery; 215: 326–331.*

**This study was designed to investigate issues concerning "in-apparent carcinoma" of the gallbladder and the effectiveness of a radical second operation in the treatment of inapparent carcinoma. Ninety-eight patients with inapparent carcinoma were analyzed according to the "pT" category of TNM (tumor, nodes, and metastases) classification. Eighty patients underwent cholecystectomy alone, and 14 patients had a subsequent radical operation. After cholecystectomy alone it was found that (1) Patients with pT1 cancer had a 5-year survival rate (5ysr) of 100%; (2) In patients with pT2, 5ysr was 40%; and (3) Patients with pT3 showed 5ysr of 0%. Results of a radical second operation showed that (1) Patients with pT2 cancer showed a 5ysr of 90%, significantly better ($p < 0.05$) than pT2 treated with cholecystectomy alone; (2) There was a prolongation of survival in patients with pT3 or pT4. It was concluded that a radical second operation should be carried out for pT2 or more advanced inapparent carcinoma, whereas follow-up without a second operation is recommended for pT1 cancer without positive margin.**

*Yamaguchi, K. and Tsuneyoshi, M. (1992) Subclinical gallbladder carcinoma. The American Journal of Surgery;* **163**: 382–386.

**Clinicopathologic features of 31 patients with subclinical gallbladder carcinoma were reviewed in an attempt to determine the parameters for a course of therapy. Subclinical gallbladder carcinoma was defined as a gallbladder carcinoma that was first diagnosed microscopically by surgical pathologists. Of 31 patients, there were**

**26 women and 5 men, ranging in age from 54 to 84 years (mean age: 68 years). All 31 patients had undergone cholecystectomy for presumed benign gallbladder conditions. The 31 gallbladder carcinomas consisted of 6 carcinomas limited to the mucosa or the muscle coat (m or pm) and 25 carcinomas extending into the subserosal layer with surgical margins free of malignant cells in 14 (ss ew [-]) and affected by malignant cells in 11 (ss ew [+]). Cumulative 1-year, 3-year, and 5-year survival rates of six patients with m or pm carcinoma were 100% ($p < 0.001$, versus ss ew [+] at 1 year), 100% ($p < 0.05$, versus ss ew [-] at 3 years), and 100% ($p < 0.05$, versus ss ew [-] at 5 years) compared with 91% ($p < 0.01$, versus ss ew [+] at 1 year), 65%, and 65% of 14 with ss ew (-) carcinoma and 43%, 0%, and 0% of 11 with ss ew (+) carcinoma. Thirteen of the 31 patients died of local recurrence and/or liver metastasis. Univariate logrank analysis of 10 prognostic factors showed that depth of invasion, venous invasion, and surgical margin were prognostic factors. Multivariate Cox-regression analysis of these three profound factors demonstrated that surgical margin and depth of invasion were independent variables. These results showed that m or pm sub-clinical gallbladder carcinoma does not necessarily require an additional operation, whereas ss ew (-) and ss ew (+) carcinomas necessitate additional resection and adjuvant treatment.**

### PAPER DISCUSSION

KEY WORDS: Gallbladder carcinoma

Subclinical and inapparent gallbladder carcinoma is defined by both authors as being gallbladder carcinoma first diagnosed microscopically by a histopathologist. Yamaguchi and colleagues carried out a retrospective analysis of 31 patients from 16 institutions with the aim of clarifying predictive clinical factors and establishing the preferred treatment. Shirai and colleagues carried out a retrospective analysis of 98 patients from one University Hospital with the aim of defining factors which affect survival but more importantly discerning the effectiveness of a second radical operation. Several authors have recommended radical reoperation for subclinical/inapparent carcinoma of the gallbladder but no study to date has demonstrated its efficacy.

Carcinoma of the gallbladder is a relatively rare malignancy which is difficult to diagnose; extension of the disease beyond the mucosa predicts poor long term survival[1,2]. Long-term survival is typical in patients with an incidental (i.e. subclinical or inapparent) diagnosis of carcinoma of the gallbladder[3]. Between 12% and 27% of patients receiving treatment for gallbladder carcinoma undergo operation for unsuspected malignancy[4]. Establishing the diagnosis of gallbladder cancer before operation is difficult: de-Aretxabala and colleagues achieved the correct preoperative diagnosis in only one of 11 patients who had no serosal involvement, yet five of these patients had lymph node involvement and three had liver and lymphatic involvement discovered at a second operation undertaken to achieve radical local clearance[5].

Because it is known that lymph from the gallbladder drains to the pancreaticoduodenal nodes via the common bile duct and venous blood drains to the quadrate lobe of the liver, several authors have advocated radical surgery for early gallbladder cancer. Such surgery includes cholecystectomy, wedge resection of the gallbladder bed up to a depth of 2cm within the liver and resection of the supraduodenal segment of the extrahepatic bile duct and regional lymph nodes[4,5]. de-Aretxabala and colleagues have performed such a dissection in 25 patients with tumour involvement of the serosa or muscular layer only and achieved a significantly longer survival in comparison with those who had a palliative resection or simple cholecystectomy[6].

Yamaguchi and colleagues have confirmed the findings of several other studies which show that subclinical/inapparent carcinoma of the gallbladder which penetrates only the mucosa or muscle coat carries a 100% 5 year survival. Once penetration of the sub-serosal layer occurs and if in addition resection margins are positive, then one year survival decreases to only 43% compared to 91% if resection margins are negative. Recurrences and death are usually due to obstruction of the biliary tree at the hilus or remote liver or distant metastases. Of twelve patients who received adjuvant treatment (of varying type because of the multicentre nature of the study), there appeared to be no survival benefit but it may be concluded that these are small numbers and there are encouraging signs from other centres that intraoperative radiation therapy or chemotherapy may be beneficial[3,7,8]. An interesting finding in the Yamaguchi study was that 10 of the 31 patients had preoperative ultrasound findings showing marked thickening of the gallbladder wall raising the suspicion of carcinoma. In these circumstances, it would be sensible to recommend opening and inspecting the gallbladder after cholecystectomy and in selected cases, performing frozen section.

The recommendations by Shirai and colleagues that a second radical operation for (i) pT-2 carcinomas i.e. those with no extension into the serosa but which invade perimuscular connective tissue, (ii) patients with a positive resection margin and (iii) patients with a positive cystic duct lymph node are more controversial. Critical analysis of this paper reveals that the authors have given no criteria for why 14 of the 98 patients underwent a subsequent radical operation: 10 patients with pT-2 gallbladder carcinoma received a second radical operation whereas 35 received cholecystectomy alone and no selection criteria have been given for the division of the patients into two groups. Of the 10 patients receiving a second radical operation there was no residual cancer in 6, all of whom were long-term survivors. This figure is very similar to the percentage of patients with no residual lymph node involvement or liver invasion reported by de-Aretxabala and colleagues[6]. The benefit derived from such a radical resection can be questioned, particularly in the light of one death after surgery. Of the four patients with pT-2 carcinoma who did have residual cancer in the second resection specimen, two survived and two died. The authors recommendation therefore that patients with pT-2 carcinomas should receive a second radical operation is based on resection of residual cancer in only two patients who subsequently survived. It must be concluded therefore that further study of this area is warranted. Perhaps the radical reoperation was not radical enough and a block resection of the hepatoduodenal ligament with or without hemihepatectomy and pancreaticoduodenectomy is indicated[9,10].

The available evidence from Yamaguchi and Shirai is not yet firm enough to recommend a subset of patients for further radical surgery. Such a recommendation may be confounded by the fact that peritoneal seedlings may have been disseminated at the first operation negating any benefits of en bloc resection and lymphadenectomy and it may be too late to attempt clearance at a second operation[11]. Until surgeons are more alert to a preoperative or peroperative diagnosis and until the limits of radical surgery are defined the further treatment of gallbladder carcinoma invading perimuscular connective tissue but not serosa must remain an open question.

### REFERENCES

1. Paraskevopoulos, J.A., Dennison, A. and Johnson, A.G. (1991) Primary carcinoma of the gallbladder. *HPB-Surg.*, **4**, 277–289
2. Jones, R.S. (1990) Carcinoma of the gallbladder. *Surg. Clin. North Am.*, **70**, 1419–1428
3. Nadlar, L.H. and McSherry, C.K. (1992) Carcinoma of the gallbladder: review of the literature and report on 56 cases at the Beth Israel Medical Center. *Mount Sinai J. Med.*, **59**, 47–52
4. Bergdahl, L. (1980) Gallbladder carcinoma first diagnosed by microscopic examination of gallbladders removed for presumed benign disease. *Ann. Surg.*, **191**, 19–22
5. de-Aretxabala, X., Roa, I., Araya, J.C., Burgos, L., Flores, P., Huenchullan, I. and Miyazaki, I. (1990) Operative findings in patients with early forms of gallbladder cancer. *Br. J. Surg.*, **77**, 291–293
6. de-Aretxabala, X., Roa, I., Burgos, L., Araya, J.C., Fonseca, L., Wistuba, I. and Flores, P. (1992) Gallbladder cancer in Chile. A report on 54 potentially resectable tumors. *Cancer*, **69**, 60–65
7. Houry, S., Schlienger, M., Huguier, M., Lecaine, F., Perine, F. and Lugier, A. (1989) Gallbladder carcinoma. Role of radiation therapy. *Br. J. Surg.*, **76**, 448–450
8. Busse, P.M., Cady, B., Bothe, A., Jenkins, R., McDermott, W.D., Steele, G. and Stone, M.D. (1991) Intraoperative radiation therapy for carcinoma of the gallbladder. *World J. Surg.*, **15**, 352–356
9. Mimura, M., Takakura, M., Kim, H., Hamazaki, K., Tsuge, H. and Ochiai, Y. (1991) Block resection of the hepatoduodenal ligament for carcinoma of the bile duct and gallbladder. *Hepatogastroenterology*, **38**, 561–567
10. Matzumoto, Y., Fujii, E., Aoyama, H., Yamamoto, M., Sugahara, K. and Suda, K. (1992) The surgical treatment of primary carcinoma of the gallbladder based on the histologic analysis of 48 surgical specimens. *Am. J. Surg.*, **163**, 239–245
11. Pezet, D., Fondrinier, E., Rotman, N., Guy, L., Lemesle, P., Lointier, P. and Chipponi, J. (1992) Parietal seeding of carcinoma of the gallbladder after laparoscopic cholecystectomy. *Br. J. Surg.*, **79**, 230

A.N. Kingsnorth
Senior Lecturer in Surgery
Department of Surgery
The University of Liverpool
P O Box 147
Liverpool L69 3BX
United Kingdom

### SCLEROTHERAPY VERUS PROPRANOLOL AFTER A VARICEAL BLEED

### ABSTRACT

*Dasarathy, S., Dwivedi, M., Bhargava, D.K., Sundaram, K.R. and Ramachandran, K. (1992) A prospective randomized trial comparing repeated endoscopic sclerotherapy and propranolol in decompensated (Child B and C) cirrhotic patients. Hepatology, 16, 89–95.*

